# Design and Evaluation of User-Centered Exergames for Patients With Multiple Sclerosis: Multilevel Usability and Feasibility Studies

**DOI:** 10.2196/22826

**Published:** 2021-05-07

**Authors:** Alexandra Schättin, Stephan Häfliger, Alain Meyer, Barbara Früh, Sonja Böckler, Yannic Hungerbühler, Eling D de Bruin, Sebastian Frese, Regula Steinlin Egli, Ulrich Götz, René Bauer, Anna Lisa Martin-Niedecken

**Affiliations:** 1 Department of Health Sciences and Technology, Institute of Human Movement Sciences and Sport ETH Zurich Zurich Switzerland; 2 Department of Design, Subject Area in Game Design Zurich University of the Arts Zurich Switzerland; 3 Division of Physiotherapy, Department of Neurobiology, Care Sciences and Society Karolinska Institute Stockholm Sweden; 4 Technology and Innovation Unit and Department of Research, ZURZACH Care Bad Zurzach Switzerland; 5 Physiotherapy Langmatten Binningen Switzerland

**Keywords:** multiple sclerosis, exergame, motor, physical, cognition, usability, feasibility

## Abstract

**Background:**

Multiple sclerosis (MS) is a chronic inflammatory disease of the central nervous system. Patients with MS experience a wide range of physical and cognitive dysfunctions that affect their quality of life. A promising training approach that concurrently trains physical and cognitive functions is video game–based physical exercising (ie, exergaming). Previous studies have indicated that exergames have positive effects on balance and cognitive functions in patients with MS. However, there is still a need for specific, user-centered exergames that function as a motivating and effective therapy tool for patients with MS and studies investigating their usability and feasibility.

**Objective:**

The aim of this interdisciplinary research project is to develop usable and feasible user-centered exergames for the pressure-sensitive plate Dividat Senso by incorporating theoretical backgrounds from movement sciences, neuropsychology, and game research as well as participatory design processes.

**Methods:**

Focus groups (patients and therapists) were set up to define the user-centered design process. This was followed by the field testing of newly developed exergame concepts. Two sequential usability and feasibility studies were conducted on patients with MS. The first study included a single exergaming session followed by measurements. Between the first and second studies, prototypes were iterated based on the findings. The second study ran for 4 weeks (1-2 trainings per week), and measurements were taken before and after the intervention. For each study, participants answered the System Usability Scale (SUS; 10 items; 5-point Likert Scale; score range 0-100) and interview questions. In the second study, participants answered game experience–related questionnaires (Flow Short Scale [FSS]: 13 items; 7-point Likert Scale; score range 1-7; Game Flow questionnaire: 17 items; 6-point Likert Scale; score range 1-6). Mixed methods were used to analyze the quantitative and qualitative data.

**Results:**

In the first study (N=16), usability was acceptable, with a median SUS score of 71.3 (IQR 58.8-80.0). In the second study (N=25), the median SUS scores were 89.7 (IQR 78.8-95.0; before) and 82.5 (IQR 77.5-90.0; after), and thus, a significant decrease was observed after training (z=−2.077; P=.04; *r*=0.42). Moreover, high values were observed for the overall FSS (pre: median 5.9, IQR 4.6-6.4; post: median 5.8, IQR 5.4-6.2) and overall Game Flow Questionnaire (pre: median 5.0, IQR 4.7-5.3; post: median 5.1, IQR 4.9-5.3). A significant decrease was observed in the item *perceived importance* (FSS: z=−2.118; P=.03; *r*=0.42). Interviews revealed that user-centered exergames were usable, well accepted, and enjoyable. Points of reference were identified for future research and development.

**Conclusions:**

The project revealed that the newly developed, user-centered exergames were usable and feasible for patients with MS. Furthermore, exergame elements should be considered in the development phase of user-centered exergames (for patients with MS). Future studies are needed to provide indications about the efficacy of user-centered exergames for patients with MS.

## Introduction

### Background

Globally, approximately 2.3 million people have multiple sclerosis (MS) [1]. MS is an immune-mediated chronic inflammatory disease in which focal inflammation causes the degradation of myelin in the nerve fibers of the central nervous system (CNS), resulting in a wide range of symptoms and impairments [2-4]. Depending on the affected CNS regions and the degree of severity, patients with MS can have physical disabilities (eg, motor weakness, spasticity, sensory disturbances, ataxia, and visual loss), cognitive dysfunction (eg, information processing, attention, executive functions, and memory), and fatigue [5-7]. Symptoms and disabilities affect quality of life by increasing the risk of falls, mobility restrictions, and social isolation [5,6,8-14]. Moreover, patients with MS are often physically inactive or have a sedentary lifestyle as a consequence of the abovementioned symptoms and disabilities, initiating a vicious circle of deconditioning and worsening of symptoms [15,16]. MS is commonly diagnosed in young adults between 20 and 40 years of age and thus affects the early stages of their working lives [5]. All these factors lead to an increase in social and health care costs [17,18]. Therefore, there is a huge socioeconomic need to stabilize and counteract physical disabilities and cognitive dysfunctions by introducing effective therapies for patients with MS.

In general, physical exercise is a safe method that can yield beneficial effects such as depending on the training content, muscular strength, and aerobic capacity and, consequently, it improves mobility, fatigue, and quality of life in patients with MS [19-21]. A further training method that counteracts the aspect of cognitive decline is computer-based training. Specific computer-based training seems to positively influence different cognitive functions (eg, information processing, executive functions, and memory domains) in patients with MS [22-24]. However, both methods train the physical and cognitive components separately. A concurrent offering of both training components seems to be promising because this would promote the interplay of physical and cognitive functions and thus add *everyday life ecological validity* to the training approach [25]. 

An upcoming training method that concurrently combines the training of physical and cognitive functions is exergaming [26], “technology-driven physical activities, such as video game play, that require participants to be physically active or exercise in order to play the game” [27]. Typically, a player physically interacts with a video game represented on the screen via special controller technologies. Controllers track the player’s movements and mediate them into a virtual game scenario that provides audio-visual feedback. In this way, commercially available exergames (eg, Nintendo Wii, Sony Move, or Microsoft Kinect) have successfully turned living rooms into playful training settings for approximately 10 years [28,29]. Apart from the entertainment market, video game–based training and therapy applications have also established themselves in the fitness and rehabilitation industry (eg, game-based, robot-assisted movement therapy [30,31]; virtually augmented climbing [32]; or exergame fitness training [29,33,34]). Besides the various beneficial effects of exergaming [35-37], the physical-cognitive interaction of exergames seems to trigger an alternating brain-body communication. Depending on the video game stimuli and the body-controller interaction, different cognitive and physical functions can be trained, which makes exergames a promising tool in MS therapy.

In recent years, researchers have started to evaluate exergames as a rehabilitation tool for patients with MS. Exergames proved to be an acceptable, feasible, safe, enjoyable, challenging, and self-motivating tool [38-40]. Kramer et al [41] concluded that the integration of exergames seemed to have a positive effect on training adherence and therefore could support the efficacy of long-term rehabilitation. Video game–based exercises, especially Nintendo Wii Fit, seem to improve static and dynamic balance as well as gait performance in patients with MS [41-44]. Intriguingly, these exercises led to improvements in the myelin sheaths of nerves in the brain areas involved in balance and movement [45]. Robinson et al [46] showed that the physical benefits of Nintendo Wii Fit training were comparable with traditional balance training in patients with MS. Furthermore, 2 recent systematic reviews concluded that exergaming enhanced cognitive functioning, in particular decision-making processes (executive functions) and visuospatial perception, in neurological patients who experience stroke, Parkinson disease, MS, or dementia [40,47]. However, many of the results so far stem from commercially available exergame systems (mainly Nintendo Wii and fewer Xbox Kinect and Sony PlayStation) that have not been developed for specific rehabilitation audiences. A review of exergame training in patients with MS suggested the development of exergames that target the training of a clinically identifiable need for this patient group [48]. For example, Nintendo Wii games did not appear to be entirely suitable for rehabilitation in MS because of a lack of flexibility and adaptability to the needs of patients with MS, which require special software development [49].

Human-computer interaction research, sports science, and human movement sciences offer numerous guidelines and frameworks aiming for more attractive and effective full-body motion games for different target populations [28,32,50-57]. Accordingly, these games should consider the needs and constraints of the target population [55,58,59]. One of these frameworks is the dual-flow concept that requires individual adaptable training features, thus ensuring that exergames are user-centered [55]. The dual-flow approach implies that exergame-based training provides an individual and optimal level of physical and cognitive challenge for every trainee throughout each training session by adapting the difficulty and complexity of the game to an individual’s current physical, cognitive, and emotional states and needs in real time. Furthermore, the technology-based system of exergames allows the systematic and individual integration of training principles such as intensity, volume, progression, tailoring, and feedback [60-62]. Specific software algorithms continuously analyze and rate performance, thus allowing real-time adaptations. Recent findings of the international game research debate indicate that a player can be optimally motivated and stimulated with an adaptive game mechanic [29,34,55,63-65]. In combination with an audio-visually appealing exergame scenario (visuals, sound, story, etc), players’ motivation can be increased [64]. *Having fun while training* with interactive games might have a huge impact on engagement and compliance [66]. Thus, a holistic exergame design approach can achieve an attractive and effective training experience by considering the levels of body, controller, and game scenario [33,67].

### Objectives

In summary, there is a huge potential for developing effective and attractive user-centered exergames that combine training principles with elements of game design and focus on disease-specific deficits to increase motivation and performance and thus to ensure the possibility of successful training. The overall aim of the interdisciplinary research and development work presented here is to develop and evaluate user-centered exergames for the game controller Dividat Senso by incorporating a theoretical background from movement sciences, neuropsychology, and game research, as well as participatory design processes with patients with MS and their therapists. This work aims to contribute specifically to the following: (1) research-based, iterative, co-designed user-centered exergames for patients with MS and (2) the usability and feasibility testing of newly developed exergames by field testing and study trials.

## Methods

### MS Exergame Concept

#### Design Process

**Figure 1:**
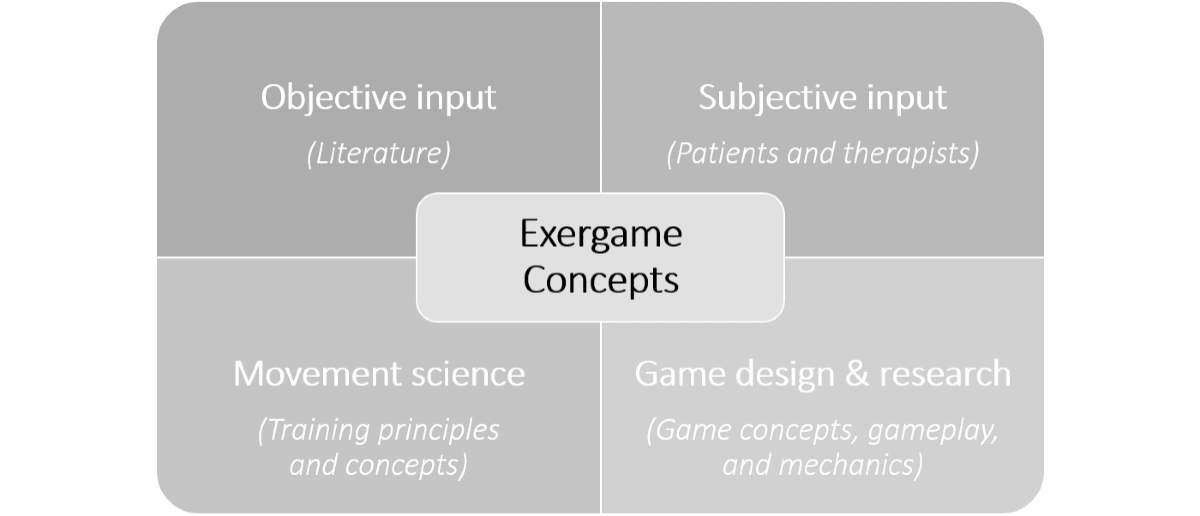
Iterative and research-based development process.

As a first step, the iterative and research-based development process of the exergame concepts considered the knowledge gained from different user perspectives (patients with MS and therapists) and disciplines (human movement science and neuropsychology as well as game design and research) to holistically generate a potentially attractive and effective user-centered exergame training for cognitive-motor therapy in patients with MS (Figure 1). The multilevel design approach covered important aspects of the exergame concept: the hardware (the Dividat Senso plate), training concept (input movements, training principles, and cognitive tasks), and software (virtual game scenario).

**Figure 2:**
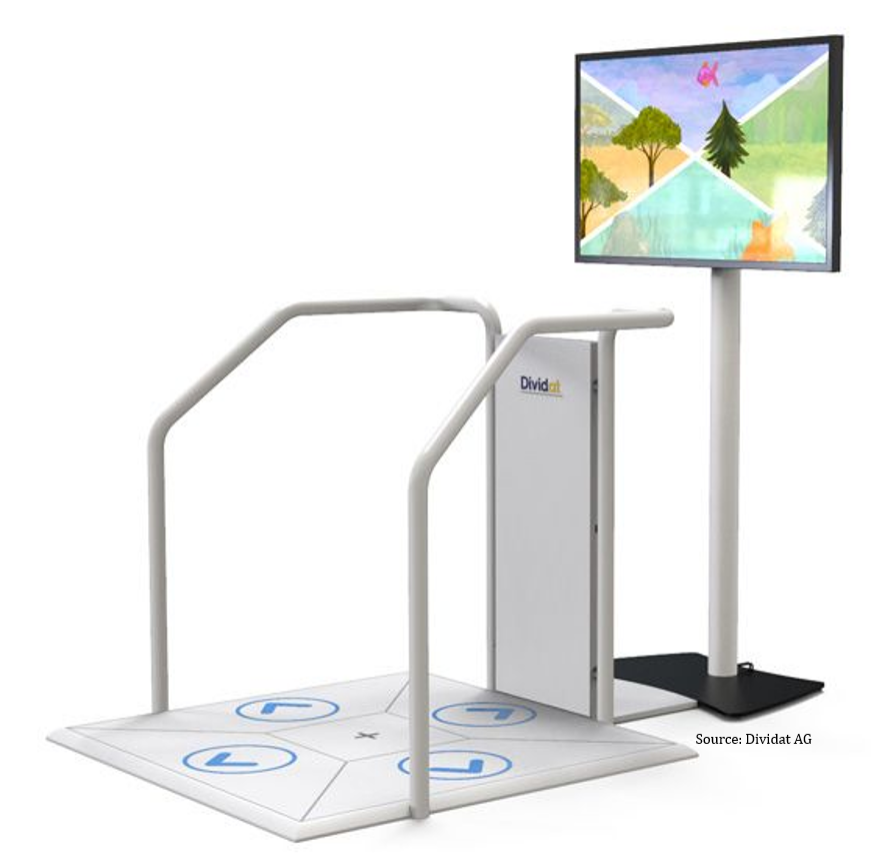
Original setup of the Dividat Senso.

This interdisciplinary research and development project developed new exergame concepts for the game controller Dividat Senso (Dividat; Figure 2). The Dividat Senso is a pressure-sensitive plate that serves as a game-input device. It uses specific lower body movements (eg, footsteps or weight shifts) to control various game scenarios presented on a screen. Several high-resolution sensors in the plate measure the force dynamically through body movements. The Dividat Senso plate further allows the generation of multidimensional sensory stimuli (eg, auditory, visual, and tactile). To support the trainee and for safety reasons, the plate is surrounded by a handrail.

#### Rethinking the Dividat Senso Plate (Hardware) and Game Designs (Software)

The design process started by analyzing the existing system and determining its technical opportunities, focusing on the Dividat Senso plate and the game collection, as they were not designed with or for the specific requirements of patients with MS. In this context, the project team visited certain therapy settings (rehabilitation center and physiotherapy) with neurologically impaired patients (MS and Parkinson disease) using the existing system in a therapy session. Furthermore, project members tested the plate and existing games themselves. 

**Figure 3:**
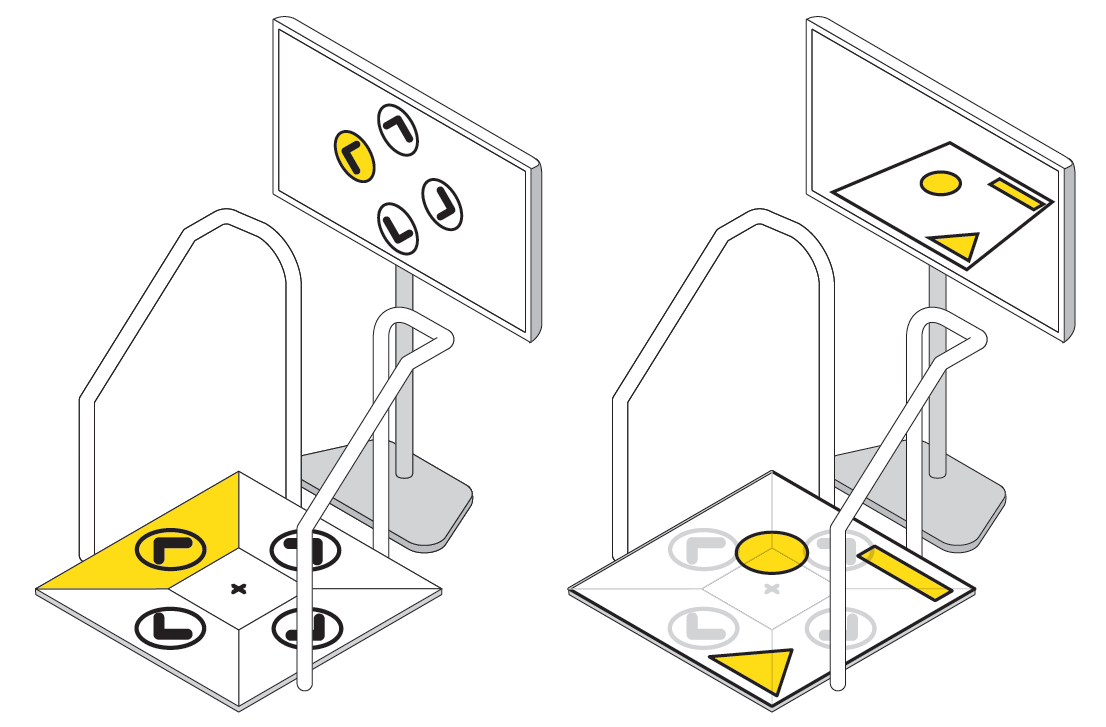
Rethinking the Dividat Senso plate. Concepts for more intuitive and natural input movements and flow are shown.

The most important finding was that patients often showed similar interaction patterns while playing on the Dividat Senso; patients first focused on the screen to receive the visual game stimuli and then tended to look down at the plate to step on the plate area to trigger the respective game input. This process seems to be important for patients with MS, as the motor learning process can be triggered via cognitive and motor information processing and realization [68]. However, the game control did not leave much room for maneuver, required very precise stepping, and did not make use of the whole plate. Such usage might interrupt movement dynamics and game flow [29,34,64,65] and leave certain gameplay options unused. Therefore, the plate layout was reconsidered, aiming for more intuitive, natural, and everyday-like patterns [34,69,70]. The focus was on using the entire pressure-sensitive plate, allowing the player to keep focusing on the game scenario and thus to stay uninterrupted in the game flow (Figure 3).

Moreover, some of the existing games did not necessarily follow a *meaningful design* [34] in terms of player perspectives [71] and the audio-visual representations of the cognitive stimuli and the respective motor challenges. For example, a virtual skier skies downhill while avoiding crashing into obstacles. The skier is represented on the screen in a third-person perspective with a top-down view and descends from above the screen but is controlled by sideways movements on the Dividat Senso plate where the left hand and right hand are flipped.

#### Rethinking the Training Concepts (Cognitive and Motor Tasks)

On the basis of the above reflections (usage and interaction patterns), the existing training concepts were also reconsidered, focusing on MS-specific motor and cognitive disabilities (eg, balance and coordination) and disease-specific deficits (eg, degeneration of myelin). Overall, the training concepts were developed and integrated by considering the following specific training principles: (1) type and specificity, (2) intensity, (3) progression, (4) variability, and (5) feedback [60-62]. Literature on exergaming in a therapeutic context was also considered [72].

**Figure 4:**
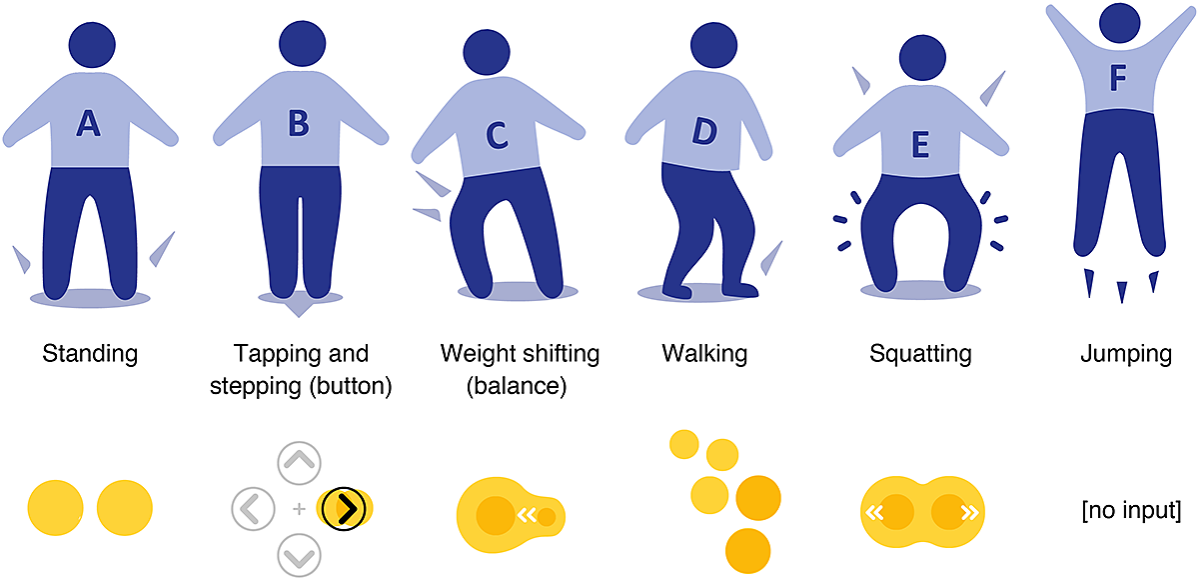
Input movements, including existing patterns (A, B, and C) and rethought patterns (D, E, and F). Input movements are presented as body models and as patterns that are registered by the pressure-sensitive plate.

In this process, some motor functions were considered that seem to be beneficial for patients with MS. Patients with MS often experience, to a variable extent, muscle weakness, diminished dexterity, spastic paresis, sensory dysfunction, gait disturbances, and fall risk, as well as fatigue and depression [5,6,73,74]. Therefore, the training concepts aimed to integrate motor control components, focusing on static and dynamic balance and coordination skills. Figure 4 shows the preexisting and reconsidered input movement. In terms of cognitive stimulation, the training concept aimed to integrate cognitive functions that may be affected in patients with MS, such as information processing, attention, decision making, error correction, executive functions, and memory [7,73-75].

A further training concept for exergames that must be mentioned is the dual-task approach. Study findings indicate that patients with MS have impaired dual- or multi-task performances that could result from their deficits in divided attention, resource capacity overload, or differential neural activation [76-80]. In this case, exergames allow the concurrent processing and synchronization of cognitive and motor stimuli and therefore seem to support constant body-brain communication. These processes might be advantageous as they are close to day-to-day activities, such as walking in an enriched real-world environment. 

Furthermore, the reconsidered training concepts considered both games that endorse motor learning [68] and games that require moderate continuous exercise performance [81] in order to replicate preliminary findings of physical training on myelin sheath regeneration as well as to specifically target important disability-related structural deficits seen in patients with MS.

#### Focus Groups: Cocreating New Exergame Concepts

**Figure 5:**
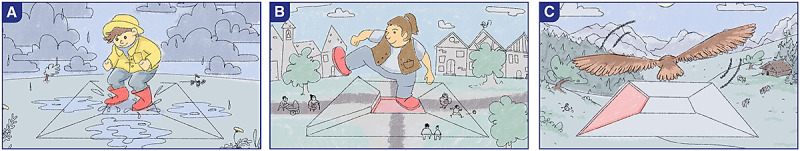
Three sketches of potential game scenarios. Different gameplay options, game mechanics, and perspectives served as inspiration during focus groups. The Puddle Jump sketch (A), the Gentle Giant sketch (B), and the Owl Flight sketch (C).

Following the rethinking process, new exergame scenarios were designed. To ensure that the concepts were user-centered, the target group (patients with MS and their therapists) was involved from the outset. A semistructured interview guideline was developed based on questions about all elements of the exergame environment (eg, body, controller, and virtual game scenarios). The aim of the focus group interviews was to explore the target group’s experiences with exergames and technology in the context of therapy, as well as to define needs, preferences, and expectations for an optimal exergame setup and its integration into an MS therapy setting. The focus group surveys took approximately 90 minutes and were carried out with 4 physiotherapists experienced in MS therapy, 9 patients with MS, and 2 specialists in neuropsychology. In addition to a list of specific questions, participants’ thoughts and specific wishes for the look and feel of future exergames were assessed using 3 different sketches of potential game scenarios (Figure 5).

On the basis of the results of the focus groups, personas for the 2 target audiences were developed. The primary aim was to provide patients with MS (predominantly adult females of all ages, ranging from high to low fitness) an attractive and effective training. The secondary aim was to provide physiotherapists (who are open to the use of technology in movement therapy) with a flexible supplementary tool to their traditional therapy methods. Among other outcomes, the focus groups revealed that the design should not be restricted to a specific age or gender group nor to a single game style and input movement concept, because the MS disease pattern is very heterogeneous. Therefore, different exergame scenarios were designed, including different game mechanics, narratives, perspectives, and input movements with the Dividat Senso. Each scenario provided slightly different cognitive and motor challenges and aimed at patients with MS aged around 30-85 years who fulfilled further requirements (see the study criteria in *Recruitment and Participants*).

#### Field Research: Initial Concept Testing

**Figure 6:**
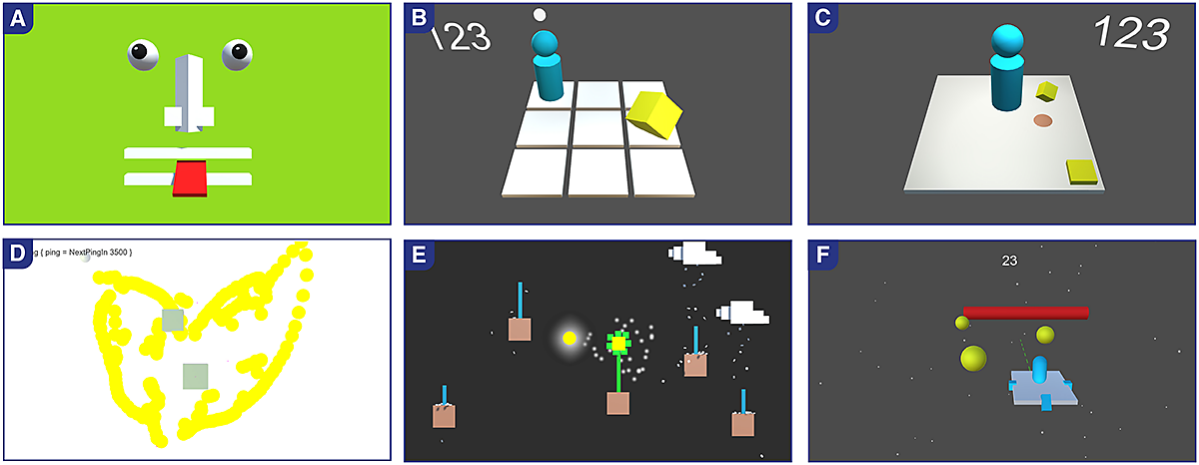
Unity 3D box prototypes. Based on the input from the focus groups, different game scenarios and mechanics were designed. A and D: Two playful, toy-like 2D prototypes allowing the feet to move freely on the Dividat Senso plate to draw and play with a face. E: 2D scenario allowing free steps or weight shifting. B and C: Two 3D images of the Dividat Senso plate acting as a virtual playground, allowing free steps and jumps. F: 3D Racer scenario with a weight shifting input.

In total, 6 box prototypes (Figure 6) were modeled using the game engine Unity 3D and showcased at numerous neurorehabilitation trade shows. After visitors of the trade shows, especially therapists and patients, tested the box prototypes, mock-ups of different themes (street, kitchen, alpine, underwater, forest, garden, oriental, and sci-fi) were shown to them. People could rate their favorite game and choose the theme that would suit them best. Out of the 6 box prototypes, the 3 concepts that were most promising and best rated were retained. The survey showed that both patients and therapists of different gender and age groups rated natural, garden-like game settings the highest.

#### Game Concepts: Design, Redesign, and Finalization

**Figure 7:**
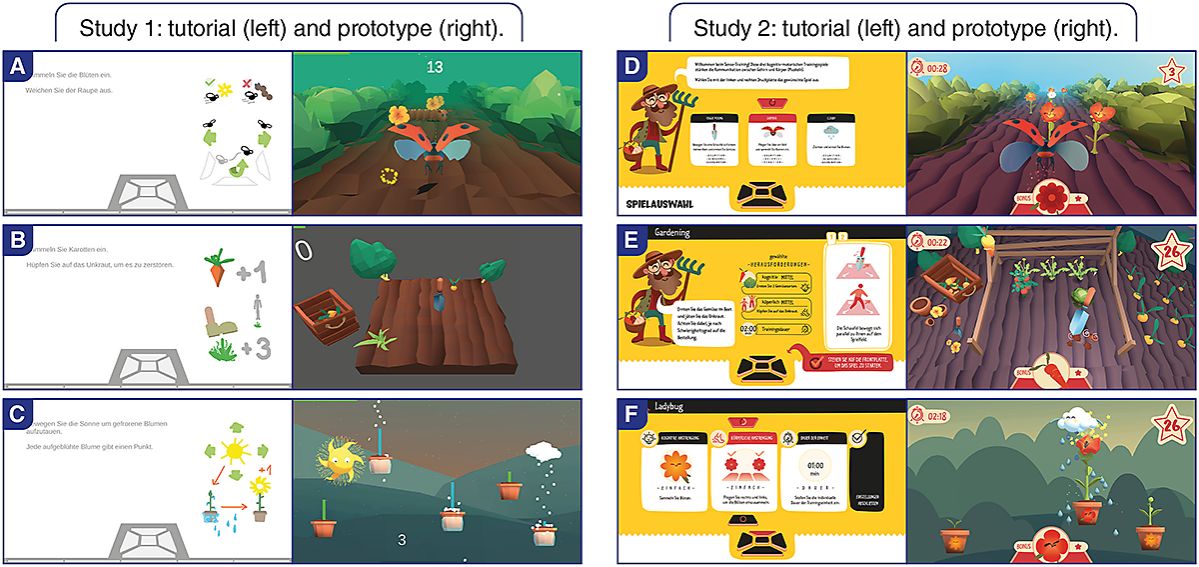
Study setup and in-game screenshots of the tutorial and game tested in the first study (A, B, and C) and in the second study (D, E, and F).

Following the preliminary field research, 3 exergame concepts were designed, including different virtual game scenarios and game mechanics, each demanding other input movements on the Dividat Senso plate. The specific descriptions of the video games, visualization of the input movements, and visual progression overview can be found in Table 1, Figure 4, and Figure 7, respectively. In all 3 exergames, the following training principles of motor learning [68] were integrated specifically to train MS-specific disabilities (eg, balance and coordination) and disease-specific deficits (eg, degeneration of myelin): (1) type and specificity (MS-specific motor and cognitive components; see also *Rethiking the Training Concepts* and Table 1); (2) intensity and progression (level adjustment and in-exergame adaptation [movement speed avatar, Ladybug] allowing for moderate continuous exercise experiences) [81]; (3) variability (3 exergames to capture different training foci; Table 1); and (4) feedback (scoring and sound effects).

**Table 1:** Game concepts for the game controller Dividat Senso.

Exergames	Ladybug	Scooper	Cloudy
Description	Navigation of a ladybug to collect randomly allocated flowers and avoid collisions with obstacles	Harvesting garden vegetables	Setting the position of the sun (Study 1) or a rain cloud (Study 2) to grow flowers
Motor components	Static balance and coordination	Dynamic balance, coordination, accuracy, and strength	Static balance, coordination, accuracy, and strength
Cognitive components	Information processing, anticipation, selective attention, and visual-spatial orientation	Information processing, planning, selective attention, and visual-spatial orientation	Information processing and selective attention
Motor-level settings (Study 2)	Level 1: Side stepping, tapping or weight shifting Level 2: Side stepping, tapping or weight shifting and stepping to the front to avoid obstacles (stones) Level 3: Side stepping, tapping or weight shifting and stepping to the front to avoid obstacles (caterpillars)	Level 1: Walking and standing on objects for collection Level 2: Walking and squatting on objects for collection Level 3: Walking and jumping on objects for collection	Level 1: Side stepping or tapping Level 2: Side stepping or tapping and squatting to make the cloud rain Level 3: Side stepping or tapping and jumping to make the cloud rain
Cognitive-level settings (Study 2)	Level 1: Pick all flowers Level 2: Pick bonus flower (2 colors) Level 3: Pick bonus flower (3 colors)	Level 1: Pick all vegetables Level 2: Pick bonus vegetables (2 colors) Level 3: Pick bonus vegetables (3 colors)	Level 1: Water all flowers Level 2: Water bonus flower (2 colors) Level 3: Water bonus flower (3 colors)

### Study Design

**Figure 8:**
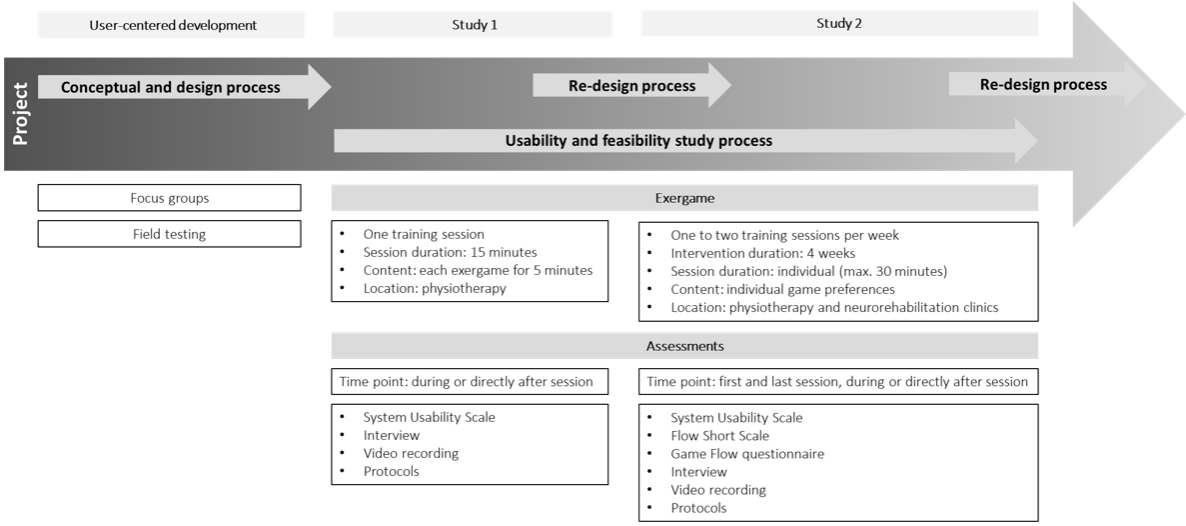
Project schedule.

Two usability studies were conducted to evaluate the usability and feasibility of the newly developed user-centered exergames in patients with MS. From January to February 2019, the measurements for the first study were taken, and from April to May 2019, the training sessions and measurements for the second study were conducted. Figure 8 shows the project process, including the 2 user studies.

In the first study, patients with MS tested each exergame concept (Figure 7) for 5 minutes in a random order. Video recordings and observation protocols for exergame performance and interaction were assessed by trained study investigators at a physiotherapy center (Physiotherapy Langmatten, Binningen, Switzerland). After the exergame sessions, patients rated the System Usability Scale (SUS) and answered predefined interview questions. 

In the second study, patients with MS played the redesigned exergame concepts (Figure 7) over a period of 4 weeks. Each patient was trained 1 to 2 times per week at a physiotherapy center (Physiotherapy Langmatten) or at one of the neurorehabilitation centers (ZURZACH Care, Rehaklinik Bad Zurzach, Bad Zurzach, Switzerland, and Reha Rheinfelden, Rheinfelden, Switzerland). In the first training session, the participants tested all 3 exergame concepts at level 1 for motor and cognitive adaptations. In the following sessions, patients could decide which exergames they wanted to play and for how long. This procedure was chosen to obtain an impression of the patient’s preferences. Regarding training progression, levels for motor and cognitive functions were individually adapted from session to session, aiming for moderate training intensities (values between 3 and 4 on the modified Borg scale, which ranges from 1 to 10) over 4 weeks. Furthermore, training time was individually increased from week to week for each patient while ensuring a minimum training time of 20-25 minutes per session. However, as the daily state of patients with MS was unpredictable, the level and training time fluctuated in some cases. In the last training session, each patient replayed each of the exergame concepts by starting from where they had left off at the last training session to familiarize themselves with the concepts before the postmeasurements. Measurements were taken during the first training session and at the last training session. During the exergame performance, video recordings and observation protocols for exergame performance and interaction were assessed. After the exergame performance, patients rated the SUS and answered the Flow Short Scale (FSS), Game Flow questionnaire, and predefined interview questions. The training sessions were supervised by trained researchers and physiotherapists, and the measurements were taken by trained researchers. 

The ethics committee of ETH Zurich, Switzerland, approved both study protocols (EK 2018-N-85 and EK 2018-N-124). Before any measurements were taken, all eligible patients provided written informed consent according to the Declaration of Helsinki. Withdrawal for no stated reason was permitted at any time during the study.

### Recruitment and Participants 

In the first study, potential participants were recruited by physiotherapists from a physiotherapy center (Physiotherapy Langmatten). In the second study, participants were recruited by physiotherapists and study investigators from specialized centers for neurological physiotherapy (Physiotherapy Langmatten) and rehabilitation (ZURZACH Care, Rehaklinik Bad Zurzach and Reha Rheinfelden). In both studies, all interested patients were fully informed about the study procedure and the inclusion criteria by physiotherapists and study investigators before screening. Patients who met the initial eligibility criteria and signed the informed consent form participated in a personal interview to screen for mental and physical health. Screened data included demographic data and medical information regarding MS (eg, MS type, leg spasticity, and fatigue). Furthermore, the following 2 questionnaires were assessed to define prevalent MS-related restrictions: the MS Impact Scale [82] and Activities-specific Balance Confidence scale [83].

For the first and second study, the same eligibility criteria were set. Patients fulfilling all the following inclusion criteria were eligible: (1) female or male; (2) aged 25-80 years; (3) clinical diagnosis of MS, including all forms (relapsing or remitting, primary-progredient, secondary-progredient, and progressive-relapsing); (4) stationary and ambulant; (5) able to provide written informed consent and understand instructions; (6) able to stand at least for 10 minutes with the aid of a handrail; and (7) visual acuity including correction sufficient to work on a television screen. Any of the following criteria led to exclusion: (1) conditions that precluded stepping exercise (severe spasticity that prevents a person from taking a full step or severe musculoskeletal injury), (2) excessive fatigue that prevented training participation, and (3) exercise intolerance that prevented training participation.

### Assessments 

Table 2 illustrates the assessments used for the first and second studies. 

**Table 2:** Study assessments.

Category			Explanation
**Feasibility**			
	Training adherence and attrition rate	Compliance with training sessions Participants lost at follow-up (dropouts)	
**Usability**			
	System Usability Scale	Reliable and valid tool providing a global view of subjective usability [84-86] A score of at least 70 for an “acceptable” solution, below 50 is “unacceptable,” and 50-70 is “marginally acceptable” [86] 10 items (5-point Likert Scale), score range 0-100	
	Flow Short Scale	Used to retrospectively get a typical flow-score for specific kinds of actions or situations [87] 13 items (7-point Likert Scale), score range 1-7 Dimensions: flow (items 1-10), fluency (item 2, 4, 5, 7, 8, and 9), absorption (items 1, 3, 6, and 10), and perceived importance (items 11-13)	
	Game Flow questionnaire	Derived from the Sweetser and Wyeth [53] “Game Flow” model, which determines the key elements of player enjoyment 17 items (6-point Likert Scale), score range 1-6 7 main items (items 1-7) building the dimension Game Flow and 10 additional explorative exergame-specific items (items 8-17)	
**Feasibility and usability**			
	Guideline-based interview	Qualitative evaluation of the user’s game play experiences Categories: (1) overall experience, (2) game scenario, (3) Dividat Senso plate (game controller), (4) body and mind, (5) motivation, (6) training, (7) comparison to conventional movement therapy, and (8) others	
	Video recording and monitoring protocol	Exergame performance Same categories as for the interview	
**Training parameters**			
	Physical and cognitive exertion	Modified Borg Scale from 1 to 10 [88]	
	Number of trainings	Range from 4 to 8 trainings	
	Training time	How long participants trained per session	
	Play preferences	How often each exergame was played	

### Data Analysis

For quantitative data, statistical analysis was conducted using SPSS (IBM SPSS 26). The level of significance was set at P<.05. The data were compared using the Wilcoxon signed-rank test, as the assumptions for parametric statistics were not met (nonnormally distributed data). The effect size (*r*) was calculated using the following equation [89]:

*r* = z/√ (N)

An effect size of 0.10–0.29 indicates a small effect, an effect size of 0.30–0.49 indicates a medium effect, and *r*≥0.50 indicates a large effect [89]. The interviews were assessed by 5 of the authors (1 game researcher and 4 movement scientists) following an iterative thematic coding approach based on qualitative content analysis [90]. For all interviews, the coders individually transcribed and coded the data according to the categories of the interview guidelines. In 2 iterations, the coders discussed the emerging results until an agreement was reached. Finally, two of the authors (1 game researcher and 1 movement scientist) further summarized the findings. A preliminary explorative analysis was conducted on the observation protocols and videos, but for the purpose of this paper, they were only used to check certain findings from the interview analysis. 

## Results

### Participants

The participant characteristics are shown in Table 3. At the beginning of the second study, 29 patients with MS were included, while 4 patients with MS dropped out (attrition rate: 4/29, 14%) during the study period. The reasons for dropout were disease-related weakness, physical condition, early clinical release, and scheduling conflicts. In total, participants completed 70 training sessions (mean 4.8 training per participant, SD 1.1) with the exergames. Of the 25 patients with MS, 4 patients with MS missed a training session once and 1 patient with MS missed a training session twice, leading to an attendance rate of 95% (120/126). The reasons for missed training were overload and fatigue after training, illness, absence, date conflict, and holiday. Considering the game preferences in the second study, the participants mostly played *Ladybug* (1.51 sessions per training), followed by *Scooper* (1.19 sessions per training), and the least *Cloudy* (0.91 sessions per training). Overall, no adverse events were recorded in the first and second studies. 

**Table 3:** Baseline and training data characteristics.

Characteristics			Study 1 (N=16)		Study 2 (N=25)
**Gender, n (%)**					
	Female	10 (62)		15 (60)	
	Male	6 (38)		10 (40)	
Age (years), mean (SD)			62.1 (13.0)		57.3 (11.2)
**Types of MS^a^** **, n (%)**					
	RR^b^	7 (44)		11 (44)	
	SP^c^	2 (12)		10 (40)	
	PP^d^	7 (44)		3 (12)	
	Not applicable	0 (0)		1 (4)	
**Therapy stay, n (%)**					
	Ambulant	16 (100)		19 (76)	
	Stationary	0 (0)		6 (24)	
Diagnosis since (years), mean (SD)			22.73 (13.1)		16.6 (11.7)
MSIS^e^, mean (SD)			33.2 (16.7)		34.3 (15.0)
MSIS physical, mean (SD)			34.2 (20.1)		36.2 (15.5)
MSIS psychological, mean (SD)			29.7 (23.0)		32.1 (19.2)
ABC^f^, mean (SD)			74.7 (11.7)		69.4 (18.2)
Exergame experience, n (%)			2 (13)		10 (40)
Number of trainings per participant, mean (SD)			1 (0)		4.8 (1.1)
Training time per session (min), mean (SD)			15 (0)		19.1 (3.9)
Borg motor, mean (SD)			3.3 (1.2)		3.8 (1.8)
Borg cognitive, mean (SD)			4.0 (1.8)		3.5 (1.9)

^a^MS: multiple sclerosis.

^b^RR: relapsing-remitting.

^c^SP: secondary-progressive.

^d^PP: primary-progressive.

^e^MSIS: multiple sclerosis impact scale.

^f^ABC: Activities-specific Balance Confidence scale.

### Quantitative Data

In the first study, the median SUS score was 71.3 (IQR 58.8-80.0). The SUS and questionnaire pre-post comparisons of the second study are presented in Table 4.

**Table 4:** Questionnaire data (N=25).

Questionnaires^a^			Pre, median (IQR)		Post, median (IQR)		z		P value		*r*
System Usability Scale			89.7 (78.8-95.0)		82.5 (77.5-90.0)		−2.077		.04^b^		0.42
**Flow Short Scale^c^**			5.9 (4.6-6.4)		5.8 (5.4-6.2)		−0.400		.69		0.08
	Fluency	5.7 (4.4-6.6)		5.6 (4.8-6.6)		−0.325		.75		0.07	
	Absorption	5.8 (5.1-6.5)		6.0 (5.1-6.6)		−0.485		.63		0.10	
	Perceived importance^d^	2.0 (1.5-3.8)		1.3 (1.0-3.5)		−2.118		.03^b^		0.42	
**Game Flow^c^**			5.0 (4.7-5.3)		5.1 (4.9-5.3)		−0.473		.64		0.09
	Concentration	5.0 (5.0-6.0)		6.0 (5.0-6.0)		−0.775		.44		0.16	
	Challenge	4.0 (2.5-4.5)		4.0 (3.0-4.8)		−0.210		.83		0.04	
	Skills or abilities	5.0 (4.0-5.0)		5.0 (4.0-5.0)		−0.277		.78		0.06	
	Control	5.0 (4.5-5.0)		5.0 (4.5-6.0)		−0.732		.46		0.15	
	Aim	6.0 (6.0-6.0)		6.0 (6.0-6.0)		−0.816		.41		0.16	
	Feedback	6.0 (5.0-6.0)		6.0 (6.0-6.0)		−1.030		.30		0.21	
	Immersion	5.0 (5.0-6.0)		5.0 (5.0-6.0)		−0.811		.42		0.16	
	Pleasure and liking	6.0 (5.0-6.0)		6.0 (5.0-6.0)		−0.264		.79		0.05	
	Dual flow over—challenge^d^	1.0 (1.0-2.5)		1.0 (1.0-2.0)		−0.577		.56		0.12	
	Dual flow under—challenge^d^	1.0 (1.0-3.0)		2.0 (1.0-2.8)		−0.418		.68		0.08	
	System control	5.0 (4.3-5.0)		5.0 (5.0-6.0)		−1.604		.11		0.32	
	Movement	5.0 (5.0-6.0)		5.0 (5.0-6.0)		−0.351		.73		0.07	
	Motivation	6.0 (5.0-6.0)		6.0 (5.0-6.0)		−0.816		.41		0.16	
	Physical exertion^e^	4.0 (2.0-5.0)		4.0 (2.0-5.0)		−0.158		.88		0.03	
	Cognitive exertion^e^	3.0 (2.0-4.5)		3.0 (2.0-4.0)		−0.042		.97		0.01	
	Optimal challenge	5.0 (4.0-5.0)		4.0 (4.0-5.0)		−0.842		.40		0.17	
	Spatial presence	5.0 (3.5-6.0)		5.0 (4.0-6.0)		−0.361		.72		0.07	

^a^Data were analyzed using Wilcoxon signed-rank test.

^b^P<.05.

^c^The higher the scores, the better the results. This counts for all items that are not specifically marked.

^d^The lower the scores, the better the results.

^e^The more in the middle field, the better the results.

### Qualitative Data

**Figure 9:**
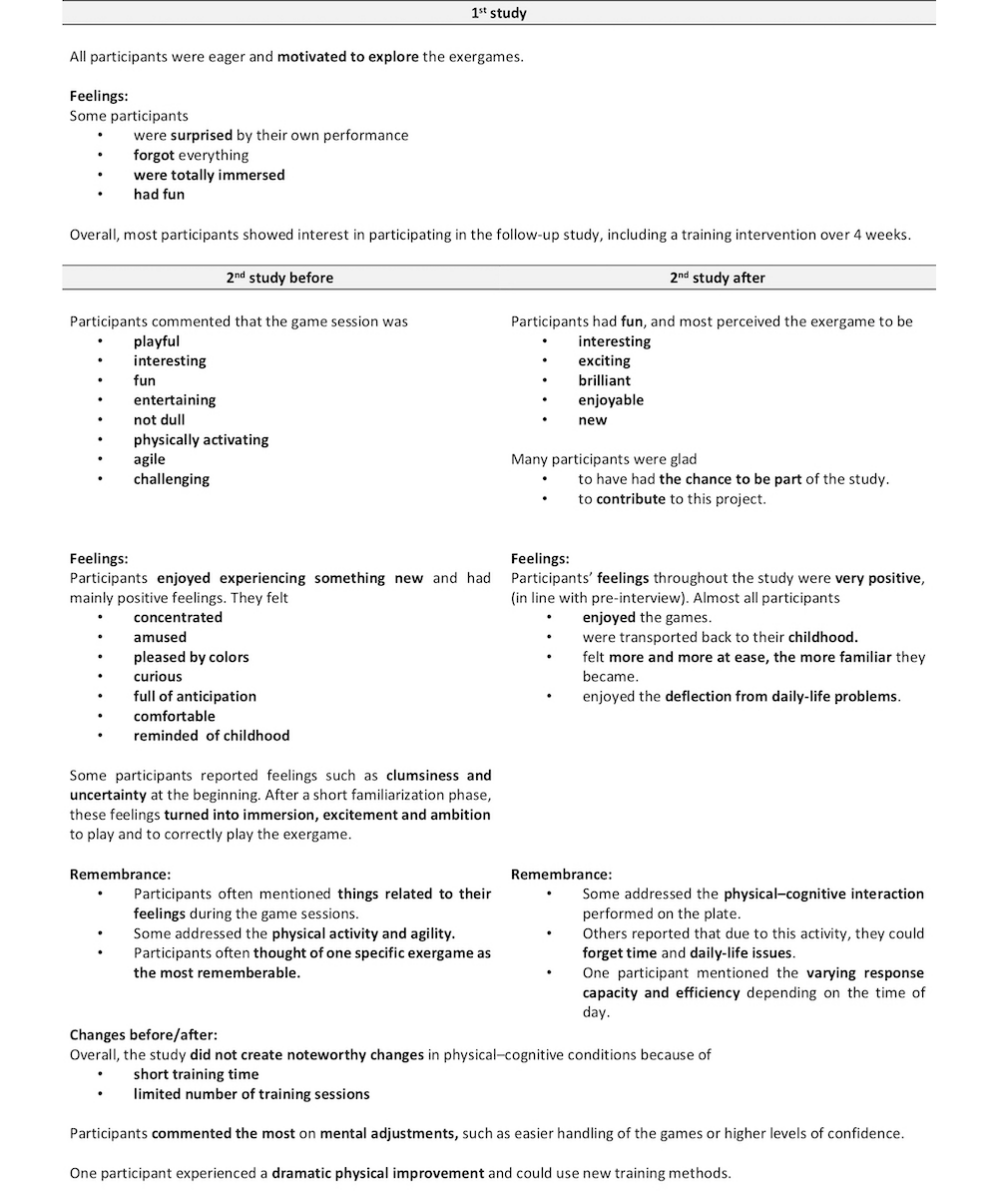
Interview data focusing on overall experience. (Some and minority = at least 30% of the participants; many = at least 50% of the participants; most and majority = at least 80% of the participants).

**Figure 10:**
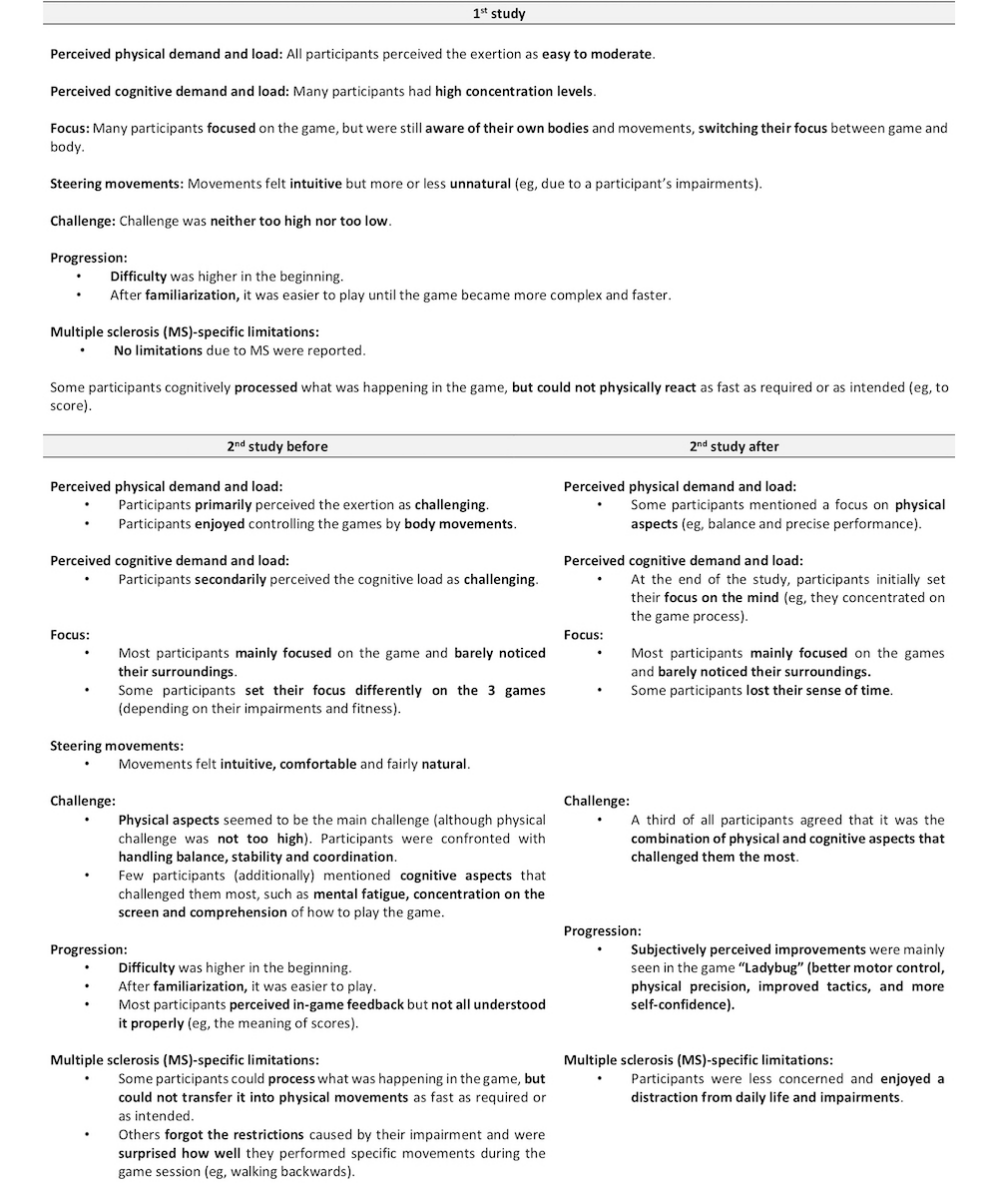
Interview data focusing on body and mind. (Some and minority = at least 30% of the participants; many = at least 50% of the participants; most and majority = at least 80% of the participants).

**Figure 11:**
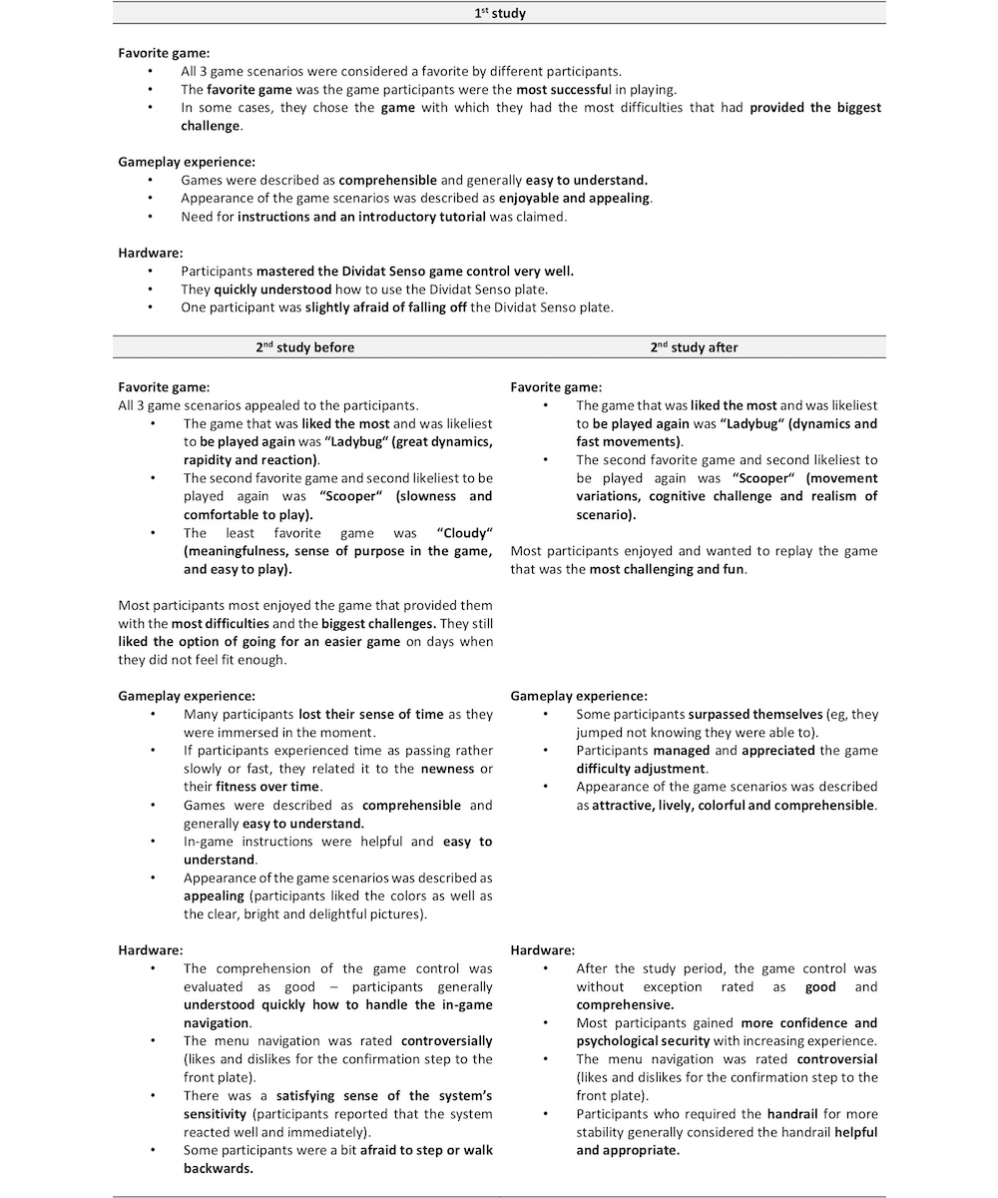
Interview data focusing on games, gameplay experience, and hardware. (Some and minority = at least 30% of the participants; many = at least 50% of the participants; most and majority = at least 80% of the participants).

**Figure 12:**
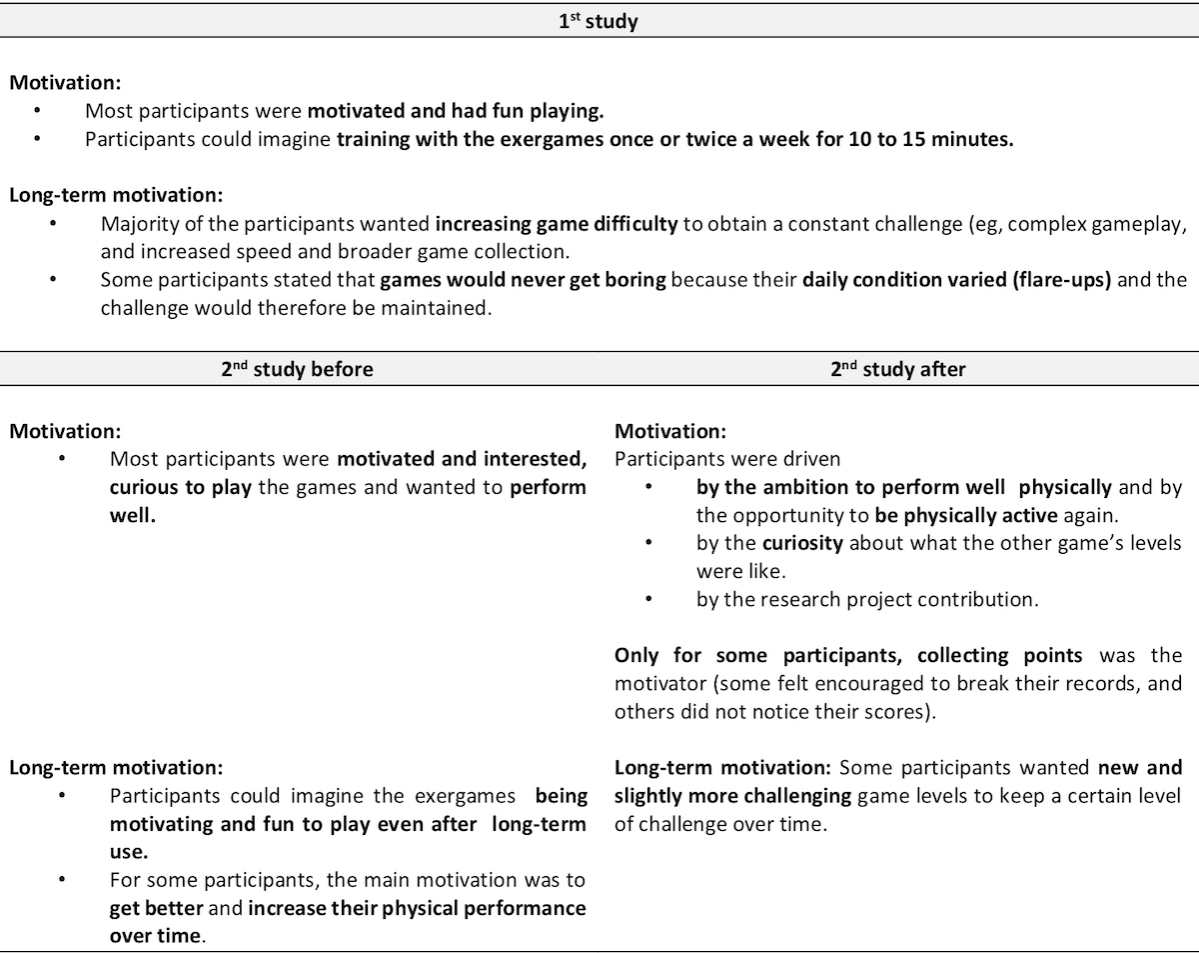
Interview data focusing on motivation. (Some and minority = at least 30% of the participants; many = at least 50% of the participants; most and majority = at least 80% of the participants).

**Figure 13:**
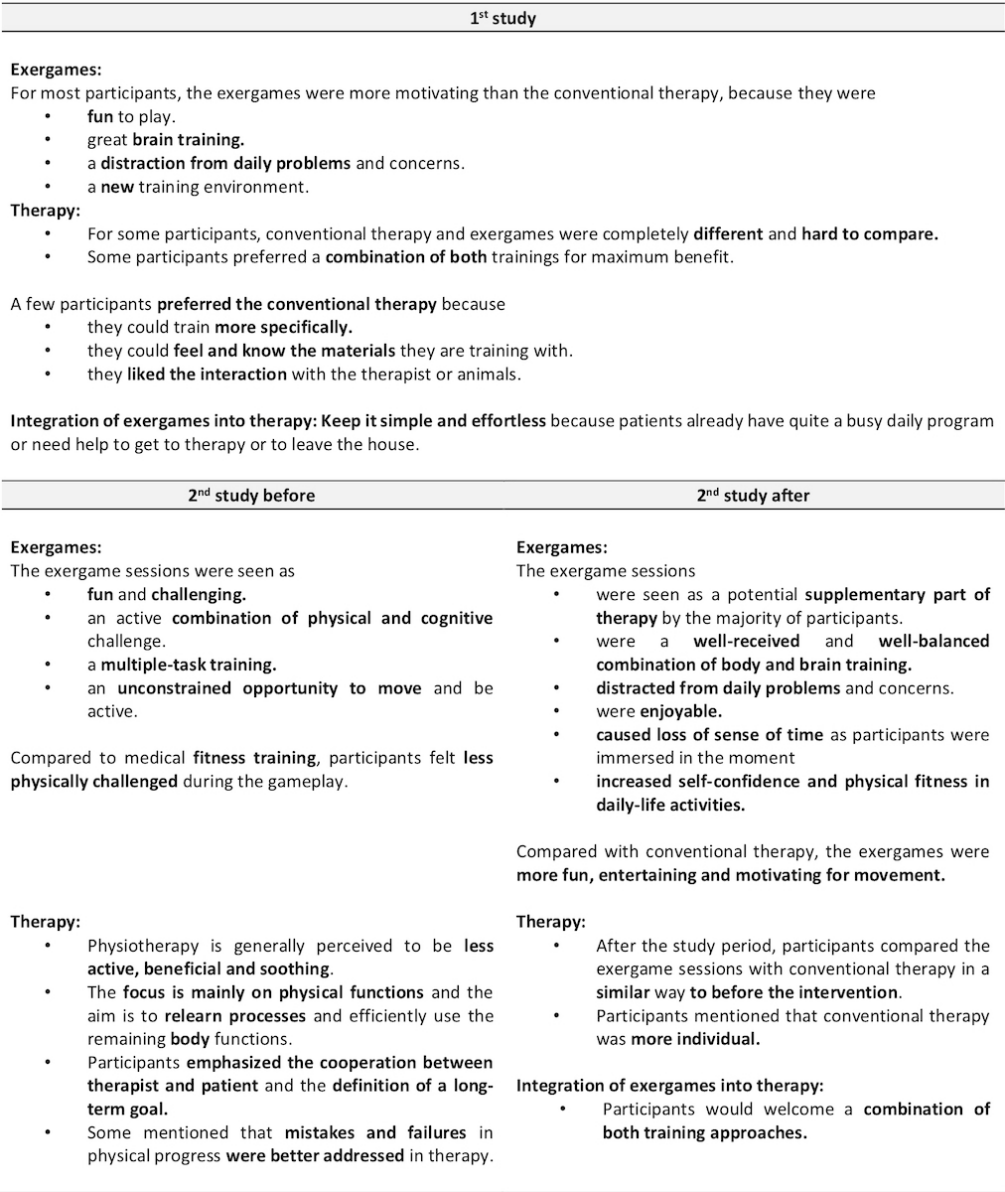
Interview data focusing on the comparison of exergames with conventional therapy. (Some and minority = at least 30% of the participants; many = at least 50% of the participants; most and majority = at least 80% of the participants).

Findings from the guideline-based interviews of both studies are reported for *overall experience* (Figure 9), *body and mind* (Figure 10), *games, gameplay experience, and hardware* (Figure 11), *motivation* (Figure 12), and the *comparison of exergames with conventional therapy* (Figure 13).

In summary, all participants reported an enjoyable, motivating, varied, and fun experience with the exergames, which was a completely new thing for most of them (Figure 9, Figure 11, and 12). They also reported that, in addition to having a lot of fun while being challenged, they felt a clear improvement in the handling (coordination and physical interaction) of the new technology over time (Figures 10 and 11), which made them feel more confident in using it (Figures 9 and 11). On the level of body and mind, participants clearly focused on the virtual gaming world, which distracted them from physical exertion and made it seem very pleasant, albeit challenging, but by no means overstraining (Figure 10). By immersing in the game world, patients were able to forget their everyday worries (often associated with the disease) for the moment (Figure 10). Regarding the potential use of exergames as a therapeutic device, most participants saw the added value of the novel training solution in terms of distraction from everyday life, fun, and the combined body and brain training approach, even though traditional therapy measures were also described very positively and were difficult to compare (Figure 13). A complementary integration of the exergames into therapy could be imagined very well by all patients. Further development of the exergames over the 2 studies was also perceived positively.

## Discussion

### Overview

This project aimed to contribute specifically to (1) develop research-based, iterative, and co-designed user-centered exergames for patients with MS and (2) determine the usability and feasibility of the newly developed exergames. This was only possible by incorporating the theoretical background from human movement sciences, neuropsychology, and game research, as well as practical skills from game design. Furthermore, this iterative and participatory design process was carried out in close collaboration with patients with MS and their therapists. 

In the following sections, the quantitative and qualitative results of the user studies are discussed and set in the context of related work and knowledge in game research and movement science, as well as research in the field of MS. Quantitative and qualitative data revealed certain exergame elements that are specific to patients with MS and can become key features for the further development of user-centered exergames for this heterogeneous target group. An outlook on future approaches in user-centered MS-specific exergame development and research will be provided.

### Shift of Focus

After the second study, patients often reported a *shift in their focus* from the physical to the cognitive level when playing exergames. Some patients even reported a shift of focus from their impairments to their actual skills and abilities, which they found to increase over the period studied. A study in older adults showed that exergame training increased the participants’ confidence and research connected this confidence with increased *self-efficacy* [91,92]. One participant could even use advanced training methods in his regular therapy at the end of the second study. The exergames allowed the patients a sense of control over their tasks, as described by Sweetser and Wyeth [53]. The more familiar participants became with the exergames and the more they trained their own gameplay strategies and body movements, the more secure, confident, immersed, absorbed, and “in the flow” they became with the exergame. The flow feeling was described not only in the interviews but also in the FSS and Game Flow questionnaire, illustrated by a high rating in several questionnaire items as well as by a significant decrease in the questionnaire item *perceived importance*. The decreased *perceived importance* item seems to indicate that the gaming challenge of the exergames was more enjoyable, as patients have attached less importance to the gaming outcomes [93]. This might have been caused by the shift of focus, the increased sense of control, the familiarization process, and higher flow feeling. Furthermore, some patients reported that gaming time distracted them from their daily-life problems and their MS-related impairments. This is in line with the findings of related studies [53,91,94].

### Heterogeneity of Patients With MS 

The heterogeneity of patients with MS, including the individual course of the disease (eg, wide range of symptoms and unpredictable flare-ups), as well as demographic details (eg, wide range of age), was also reflected in the interviews. Patients reported that game content, challenge, and progression should always be adaptable to their *individual physical, cognitive, and mental requirements and their daily form* [51,72,95]. Therefore, an exergame for patients with MS should allow an individually adaptive training focus, taking into account physical, cognitive, and mental aspects, to correspond with the heterogeneity and fluctuations of the disease pattern. The exergames covered 3 different types of game control and content; each exergame included 3 levels for motor and cognitive functions. Another relevant aspect is *security*, especially in therapeutic environments [72]. In this project, the patients could use the handrail to support exergame performance due to the insecurity of their physical stability and capacity. This security support was greatly appreciated, as presented in the results of our study. Overall, no adverse events were recorded during the entire duration of the project. The wide age range in patients with MS brings very different previous experiences in using technology [96]. Thus, even for older adult patients without previous technology use, the exergames need to be *self-explanatory and easy to use* (including help from therapists). In terms of the system’s usability in the heterogeneous study group, the iterative, participative, and interdisciplinary design process of this project was very successful as the SUS increased from study 1 to study 2. In study 2, the SUS dropped from pre- to postmeasurement. The novelty of the exergame might have distracted patients’ focus away from the usability barriers, explaining the high SUS score at the premeasurement. Furthermore, with each additional session, participants had more time to test the system and explore usability barriers. Nevertheless, the SUS in study 2 remained at a level that can be described as a usable exergame system for patients with MS [86,97].

### Training Motivation and Challenges

Most patients were motivated to train by exergames and enjoyed the requirement of physical activity for playing them. This is in line with a previous study that interviewed patients with MS about Nintendo Wii Fit [39]. However, due to the user-centered development steps and therapy focus, it may be that the training motivation was even higher than in studies that used conventional exergames [48]. One of the main *motivational drivers* was to improve the player’s body functions, to be immersed in another world, and to be distracted from daily life for the duration of the exergame session [53]. Interestingly, interviews revealed that most patients preferred more challenging games (but still not overchallenging). This was also reflected in the number of sessions in which patients chose to play the most challenging exergames. This challenging situation, in combination with the skill balance of the exergames, may have facilitated the abovementioned flow state during the training sessions [98]. Exergames should provide individually challenging but still feasible gaming experiences to increase training motivation and therefore possible training-related improvements [53]. To maintain their motivation, patients also wished for more challenging and different games or levels over time in future trials.

### Training Intensity and Progress

An exergame should be able to adapt to the individual patient at a physical and cognitive level to meet the heterogeneous and individual requirements of patients with MS and to allow for an *optimal training zone* [51,95]. For this reason, the design integrated individual levels for physical and cognitive functions into the exergames, allowing for an individually challenging game for patients with MS. To extend the playfulness and effectiveness of the exergames in the future, the assessment of certain motor and cognitive parameters (objective) or rating scales (subjective) could help to define an individual training area [99-102]. The integration of in-exergame, real-time adaptation could help to maintain a predefined optimal training zone in a training session and over a longer period (progression) [51,102,103]. In this project, participants had to rate each training session for physical and cognitive perceived exertion, allowing the training load to be adapted for the upcoming sessions. The results of the perceived exertion ratings showed that the newly developed exergames allowed for a moderate training load on the cognitive and physical levels in a single training session and over the training period. For aerobic and strength exercises, moderate training is recommended in patients with MS [104]. However, it is possible that high training intensities, such as those used in high-intensity interval training, might be even more beneficial [105]. Nevertheless, a *moderate training intensity* seems to be an appropriate approach for exergames to trigger possible motor learning processes without negatively influencing movement execution in patients with MS [68,81].

### Exergame as an MS Therapy Tool 

Interviews showed a strong acceptance of the exergames by patients (even in the first study). The majority would *welcome the integration* of exergames into their conventional therapy because of their appealing nature and beneficial motor-cognitive training approach [40,106]. The combined training regimen allows for the concurrent processing and synchronization of cognitive and motor stimuli and therefore can trigger brain-body communication. Patients with MS can have impaired dual- or multi-task performance due to possible deficits in divided attention, resource capacity overload, or differential neural activation [76-80]. Furthermore, exergames allow the integration of the patient’s conventional therapy progress in physical and cognitive functions and provide a daily-life environment in terms of the combined cognitive-motor training. However, some patients missed the *social component* and *interaction with the therapists*. Therefore, it might be interesting to specifically integrate the therapist(s) into the exergame experience by in-exergame interaction, allowing training adaptation and support. This finding is in line with recent exergame studies in patients with MS and older adults that emphasize the importance of social interaction in exergames to increase training motivation [91,103]. Moreover, social interaction is a part of the Game Flow model proposed by Sweetser and Wyeth [53]. Overall, user-centered exergames seem to be a very promising therapy tool for patients with MS, considering the abovementioned aspects of training and design principles. 

As a next step, further research and development work will deepen the knowledge of design principles in MS exergames and reveal additional insights. To meet the heterogeneous spectrum of MS and to provide an individually attractive and effective training and therapy tool, the newly developed exergames will be further iterated and extended based on the findings of the usability and feasibility studies. Furthermore, new types of use will be implemented, such as playing a multitask version of the exergames that involve upper-body input movements or sitting in a wheelchair. Moreover, further balancing game mechanics will be implemented, as well as extending the types of input, movement ranges, and tracking zone.

### Limitations

There are some limitations that can be reported for this study. In the first study, participants were trained only once with the exergames, whereas they trained multiple times in the second study. Therefore, participants might have had the chance to reflect more on and better familiarize themselves with the games in the second study, while they had only one attempt in the first study. Additionally, their feedback might have been influenced by the novelty effect. Furthermore, study testing was conducted at various clinics and institutions and it did not focus on measures of effectiveness. However, it should be emphasized that these studies should be conducted in the context of developing a complex intervention for health care settings. Within this context, intervention development contains different mandatory steps that should be taken in a sequential order [107]. In that sense, this study reflects a preintervention stage in which important principles and necessary actions for this stage were considered [107]. These findings justify continuing with studies that focus on the outputs and effects in clinical trials [107].

### Conclusions

The aim of the presented research and development work was to take the first step in the new field of user-centered exergames for patients with MS, to evaluate the usability and feasibility of the newly developed exergame concepts, to learn from the findings, and to derive design guidelines for future research and development projects in this field.

The quantitative and qualitative results of this project showed that the developed exergames were usable, feasible, well accepted, and enjoyable for patients with MS. Furthermore, the results indicated preliminary positive effects regarding the attractiveness of the newly developed, user-centered exergames. Participants enjoyed the motivating, varied, and fun experience with the exergames, which were both fun and physically as well as cognitively challenging and allowing them to forget their everyday worries (often associated with the disease) for the moment. Moreover, specific exergame elements were identified: control mechanisms through audio-visual design, adaptation of the individual difficulty level, game concept diversity addressing the patients’ heterogeneity, involvement of training principles, and considerations of the interaction of physical and cognitive impairments, especially brain-body communication.

Considering the points of discussion and design guidelines, user-centered exergames can be a promising training approach to improve physical and cognitive functions, especially brain-body communication in patients with MS. Thus, user-centered exergames might have positive effects on quality of life by reducing the risk of falling, mobility restrictions, and social isolation. Furthermore, the strengthening of body functions such as balance, coordination, and cognition seems to be a promising way to break the vicious circle of deconditioning. The evaluation of the effects of a user-centered exergame will show how far a user-centered exergame might complement or even surpass the results of conventional (exergame) approaches in patients with MS. 

## Abbreviations

CNS: central nervous system
FSS: Flow Short Scale
MS: multiple sclerosis
SUS: System Usability Scale
