# Correction: Design and Evaluation of User-Centered Exergames for Patients With Multiple Sclerosis: Multilevel Usability and Feasibility Studies

**DOI:** 10.2196/30326

**Published:** 2021-05-13

**Authors:** Alexandra Schättin, Stephan Häfliger, Alain Meyer, Barbara Früh, Sonja Böckler, Yannic Hungerbühler, Eling D de Bruin, Sebastian Frese, Regula Steinlin Egli, Ulrich Götz, René Bauer, Anna Lisa Martin-Niedecken

**Affiliations:** 1 Department of Health Sciences and Technology, Institute of Human Movement Sciences and Sport ETH Zurich Zurich Switzerland; 2 Department of Design, Subject Area in Game Design Zurich University of the Arts Zurich Switzerland; 3 Division of Physiotherapy, Department of Neurobiology, Care Sciences and Society Karolinska Institute Stockholm Sweden; 4 Technology and Innovation Unit and Department of Research, ZURZACH Care Bad Zurzach Switzerland; 5 Physiotherapy Langmatten Binningen Switzerland

In “Design and Evaluation of User-Centered Exergames for Patients With Multiple Sclerosis: Multilevel Usability and Feasibility Studies” (JMIR Serious Games 2021;9(2):e22826) the authors noted two errors.

In the originally published manuscript, the images displayed as Figures 9-13 were associated with the incorrect figure numbers. Figure captions and numbering were otherwise unaffected. The following changes have been made in the corrected version of the manuscript:

- The image displayed as Figure 9 has been corrected to appear as Figure 10.
- The image displayed as Figure 10 has been corrected to appear as Figure 11.
- The image displayed as Figure 11 has been corrected to appear as Figure 9.
- The image displayed as Figure 12 has been corrected to appear as Figure 13.
- The image displayed as Figure 13 has been corrected to appear as Figure 12.

In addition, under the section “Qualitative Data,” the following sentence appeared in the originally published version:

In summary, all participants reported an enjoyable, motivating, varied, and fun experience with the exergames, which was a completely new thing for most of them (Figures 9, 11, and 12).

This has been corrected as follows to properly format in-text references to figures according to journal style:

In summary, all participants reported an enjoyable, motivating, varied, and fun experience with the exergames, which was a completely new thing for most of them (Figure 9, Figure 11, and Figure 12).

The correction will appear in the online version of the paper on the JMIR Publications website on May 12, 2021, together with the publication of this correction notice. Because this was made after submission to PubMed, PubMed Central, and other full-text repositories, the corrected article has also been resubmitted to those repositories.

